# Effectiveness and Safety of Moxibustion on Constipation: A Systematic Review and Meta-Analysis

**DOI:** 10.1155/2020/8645727

**Published:** 2020-07-29

**Authors:** Fang Yao, Yang Zhang, Xiaohong Kuang, Qi Zhou, Lihua Huang, Jiazhu Peng, Shizheng Du

**Affiliations:** ^1^Department of Nursing, Zhangjiagang TCM Hospital Affiliated to Nanjing University of Chinese Medicine, Suzhou, China; ^2^School of Nursing, Nanjing University of Traditional Chinese Medicine, Nanjing, China

## Abstract

**Aim:**

This study aimed to evaluate the effects and safety of moxibustion in the management of constipation.

**Background:**

Constipation is extremely common in clinical practice and causes physical and mental pain to patients. This study aimed to evaluate the effects and safety of moxibustion in the management of constipation.

**Methods:**

Studies on moxibustion for constipation published up to November 2019 were searched in PubMed; EBSCO; EMBASE; Cochrane Library; and three Chinese databases, namely, China National Knowledge Internet, Wanfang, and China Biomedical Network. The methodological quality of the included studies was assessed on the basis of the CLEAR NPT system evaluation methods of Boutron. Further meta-analysis was performed using the RevMan 5.3 and Stata 15.0 software.

**Results:**

Ten randomized controlled trials involving 760 patients were included in this study. The meta-analysis revealed that, in comparison to western medicine treatment or other Chinese medicine methods (control group), moxibustion (experimental group) had a higher total effective rate (RR = 1.30, 95% CI [1.21, 1.40], *P* < 0.00001), and the clinical effectiveness of the experimental group was higher than that of the control group in any subgroup analysis. The first defecation time of the experimental group was shorter than that of the control group (SMD = −1.36, 95% CI [−2.03, −0.68], *P* < 0.0001). The clinical symptom score of the patients in the experimental group was lower than that in the control group (SMD = −0.65, 95% CI [−1.00, −0.30], *P*=0.0003). The patients in the experimental group had higher scores on Bristol stool form scale than those in the control group (MD = 0.99, 95% CI [0.48, 1.50], *P*=0.0001). However, there was no obvious difference in safety between the two groups (RR = 0.38, 95% CI [0.01, 11.8], *P*=0.58).

**Conclusions:**

Moxibustion may have better effect than other treatments on constipation. However, it is not yet possible to assess the safety level of moxibustion therapy, and the quality of the included literature is low, so rigorous studies are warranted. *Implications for Nursing and Health Policy*. The focus of this study was to evaluate the effectiveness and safety of moxibustion therapy in constipation. This evaluation showed that moxibustion therapy has a good effect on constipation and provides an effective treatment for constipation patients. Whether moxibustion therapy can be used for different syndrome types deserves further discussion.

## 1. Background

Constipation [1] is a condition characterized by prolonged periods of stool remaining in the intestine, reduced number of bowel movements, and dry stool which is difficult to discharge. A reduced number of bowel movements refers to less than three per week. Difficulties in defecation include straining in defecation, difficulty in discharge, inconvenience in defecation, time-consuming defecation, and the need for assistance with defecation [2, 3]. Clinically, bed rest, reduced activity, application of drugs, pain, bowel habits, position changes, and psychology are factors that have greatly increased the likelihood of constipation in certain disease populations [4]. The incidence of constipation in patients with stroke is 30%–60% [5–7]. Constipation is the most common complication of fracture and has a high incidence of reaching 90% [8]. Furthermore, the incidence of constipation in patients under chemotherapy is 11%–72% [9–11]. The elderly are prone to constipation due to a low amount of activity, weakness of the abdominal muscles, and slow gastrointestinal motility [12]. These findings indicate that constipation is extremely common in clinical practice. Mild constipation can result in abdominal pain, bloating, loss of appetite, and decreased sleep quality. Severe constipation can induce heart failure and even sudden death [13]. This condition increases the suffering of the patient, prolongs hospitalization period, and increases hospitalization cost. Western medicine commonly uses oral laxatives, Glycerine anal plug, and enema, which alleviate the symptoms to some extent [14] but cannot fundamentally improve the patients' intestinal function. Moreover, these management strategies increase the risk of constipation in the long term.

As one of the traditional Chinese medicine techniques, moxibustion uses a moxibustion material made of moxa. After being ignited, the moxa stick is suspended or placed in acupuncture points or lesions, causing warming. The heat of moxibustion and the drug itself achieve disease prevention and external treatment [15]. In recent years, moxibustion has been widely explored because of its safety, nontoxic side effects, simplicity, and effectiveness. Clinical reports on the treatment of constipation by using moxibustion are also increasing and show good clinical efficacy [16]. However, its treatment effectiveness and research credibility need further exploration. Therefore, a meta-analysis of published randomized controlled trials (RCTs) worldwide was conducted to evaluate the efficacy of moxibustion for constipation and provide a reliable clinical basis for moxibustion use for constipation.

## 2. Methods

### 2.1. Data Sources and Search Strategy

Studies were identified through a comprehensive search in the following databases: Pubmed, Embase, EBSCO, Cochrane Library, China Biomedical Network, China National Knowledge Internet, and Wanfang. The search time was from inception to November 2019. The search terms included “moxibustion,” “moxibustion therapy,” “constipation,” “abnormal bowel movement,” “difficulty in defecation,” and “random.”

### 2.2. Study Selection Criteria

Trials meeting all of the following criteria were included.

#### 2.2.1. Research Type

Research type included RCTs with no limitations on countries, regions, or languages.

#### 2.2.2. Research Subjects

Research subjects included patients diagnosed with constipation on the basis of the diagnostic efficacy standard of traditional Chinese medicine (TCM) disease syndromes [17] or the international Rome III diagnostic standard [18]. If an article did not adopt the above two criteria but had documents equivalent to the above criteria, the article was included in the study.

#### 2.2.3. Interventions

Patients in the experimental group all used moxibustion therapy alone. The method, time, and treatment of moxibustion were not limited to understand the general effect of moxibustion. The control group used western medicine treatment or other Chinese medicine methods except moxibustion.

#### 2.2.4. Main Outcomes

*Clinical Effectiveness Rate*. Grade information was formulated in accordance with the State Administration of Traditional Chinese Medicine's “Traditional Chinese Medicine Diagnostic Effectiveness Standards” [19] or “Guiding Principles for Clinical Research of New Chinese Medicine” [20]. According to the “Traditional Chinese Medicine Diagnosis and Efficacy Criteria,” cure is indicated as follows: defecation occurs once within two days, the stool becomes moist, the defecation is smooth, and no recurrence is observed in a short time; improvement is indicated as follows: defecation occurs within three days, the stool becomes moist, and defecation is not smooth; nonhealing is no improvement in symptoms. The “Guiding Principles for Clinical Research of Traditional Chinese Medicine New Drugs” stipulates that significant effects indicate that the frequency of defecation has returned to normal, the labor of defecation does not need to be laborious, and the shape of stool has returned to normal, manifested as formed soft stool without induration; effectiveness indicates that weekly stool has a frequency of more than two, and the defecation traits are improved; invalidity indicates that the requirements of the above standards are not met. The efficiency was calculated according to the following standards.

*First Defecation Time*. It is the time of first defecation after intervention.

*Clinical Symptom Score*. It is calculated in accordance with the “Guidelines for Clinical Research of Traditional Chinese Medicine New Drugs” for recording and comparison of symptoms before and after treatment.

*Bristol Stool Form Scale (BSS)*. The fecal properties were classified into seven categories. Level 1 (1 point): scattered lumps similar to nuts; Level 2 (2 points): sausage-shaped but lumped; Level 3 (3 points): sausage-shaped but with surface cracks; Level 4 (4 points): sausage- or snake-like, smooth, and soft; Level 5 (5 points): soft mass with clear edges; Level 6 (6 points): fleece, unclear edges, pasty stools; and Level 7 (7 points): watery stools, no solid, completely liquid.

*Side Effects*. The patient's vital signs before and after treatment and whether abdominal pain, bloating, dizziness, nausea, redness, swelling, and itching of the skin occur were observed. These symptoms were timely recorded and actively handled once they occur.

### 2.3. Study Exclusion Criteria

The exclusion criteria included the following:

1. The trial group including interventions for acupuncture
2. Repeated publications
3. Animal experiments
4. Review articles
5. Master's thesis or conference article
6. Case reports or expert experience
7. Incomplete test data or incorrect data reports

### 2.4. Data Extraction

Two researchers independently screened the articles in accordance with the established criteria for document inclusion and exclusion. The reasons for exclusion were recorded during the screening process. When the screening results of the two researchers were inconsistent, a third researcher participated in the discussion to determine whether the document would be included. The data included in the final review were independently extracted by two researchers, and the extracted contents included research, sample size, intervention measures, acupoints, intervention course, and outcome indicators.

### 2.5. Assessment of Study Quality and Reporting

Two researchers independently evaluated each literature's quality by using the CLEAR NPT system evaluation methods of Boutron [21]. The methods included seven items: (1) whether to fully follow the principle of random comparison, (2) whether the allocation concealment principle is used, (3) whether the patient is blinded and whether blinding is used in the evaluation of efficacy, (4) whether other interventions are consistent, (5) whether intentional treatment analysis is applied, (6) selective reporting, and (7) other biases. Each item can be rated as “Y,” “U,” or “N.” Disagreements between two researchers were resolved by discussion. If an agreement was still not reached, a third researcher was requested to participate in the discussion to determine the final result. Subsequently, all studies can be assigned a quality grade. A quality grade of A implied that the article fully met the above criteria and had minimum probability of occurrence of various biases. A quality grade of B indicated that the article partially met the above quality standards and that the possibility of bias was moderate. A quality grade of C indicated that the article did not meet the above quality standards at all and that the possibility of bias is high [22]. After independently evaluating the quality of the literature, two researchers held a quality discussion on each of the documents on the basis of the above evaluation criteria and reached a consensus to form a final literature quality evaluation.

### 2.6. Data Analysis

The RevMan 5.3 and Stata 15.0 software were used to analyze the extracted data. Heterogeneity test was first performed in each study. When *P* > 0.1 and *I*^*2*^ < 50%, the included studies were homogeneous, and the fixed effects model (FEM) meta-analysis was conducted. If *P* < 0.1 and *I*^*2*^ ≥ 50%, then the included studies were considered to have large heterogeneity. In this case, the source of heterogeneity should be analyzed (sensitivity analysis, subgroup analysis, and publication bias). If no evident clinical and methodological heterogeneity was observed, the random effects model (REM) was used for analysis. For continuous variables, when the measurement criteria were consistent and inconsistent, the weighted mean difference and standardized mean difference method (SMD), respectively, were used for analysis. The two categorical variables were analyzed using relative risk (RR). Each effect volume was expressed by confidence interval (CI), and *P* ≤ 0.05 was considered statistically significant.

## 3. Results

### 3.1. Searching Result

**Figure 1:**
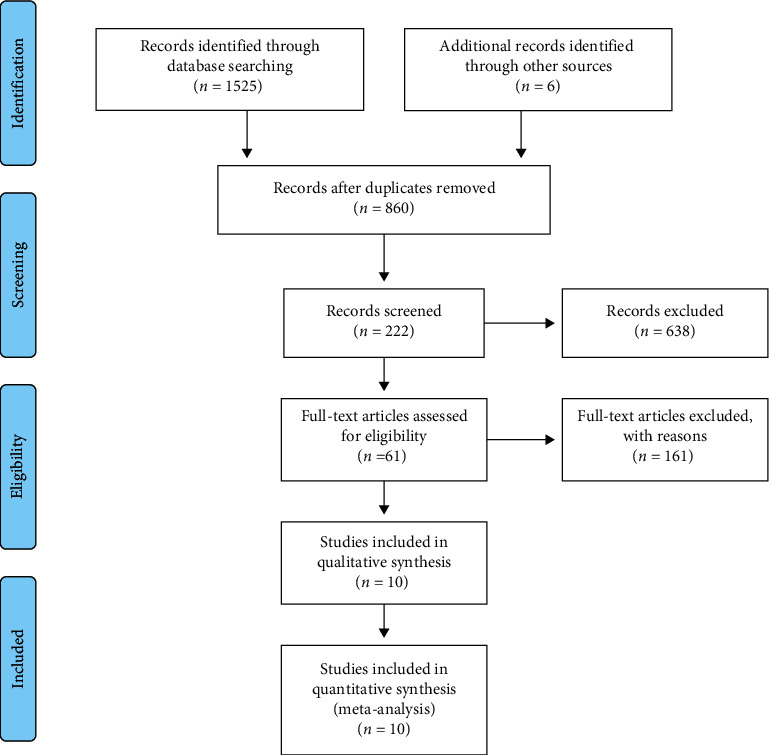
A flowchart of the characteristics of eligible articles.

A total of 1531 documents were retrieved under the search strategy (1410 and 121 articles in Chinese and English, respectively). After removing 671 duplicate articles, the title, abstract, and full text of the remaining articles were read. Finally, 10 RCTs were included [23–32]. A flowchart of the included/excluded studies is shown in Figure 1.

The detailed characteristics of the 10 articles are presented in Table 1. These studies originated from China (*n* = 9) and Korea (*n* = 1) and involved 760 patients, including 379 and 381 in the trial and the control groups, respectively. Four studies have observed adverse reactions during treatment, which have been actively addressed.

### 3.2. Quality of the Included Studies

**Figure 2:**
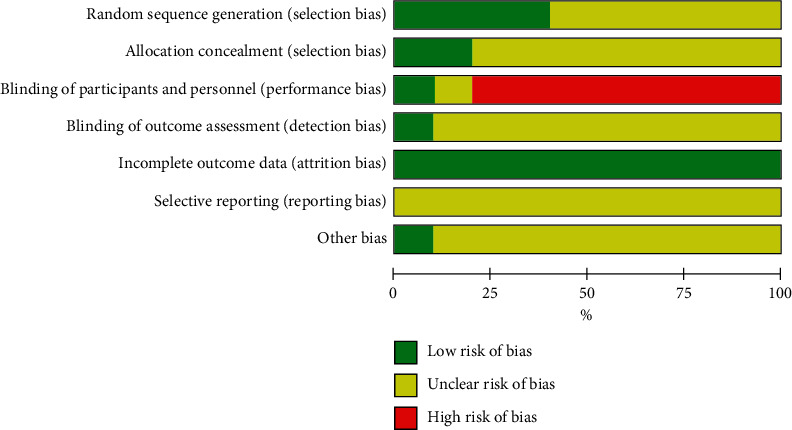
Risk of bias graph: review authors' judgments on each risk of bias item presented as percentages across all included studies.

**Figure 3:**
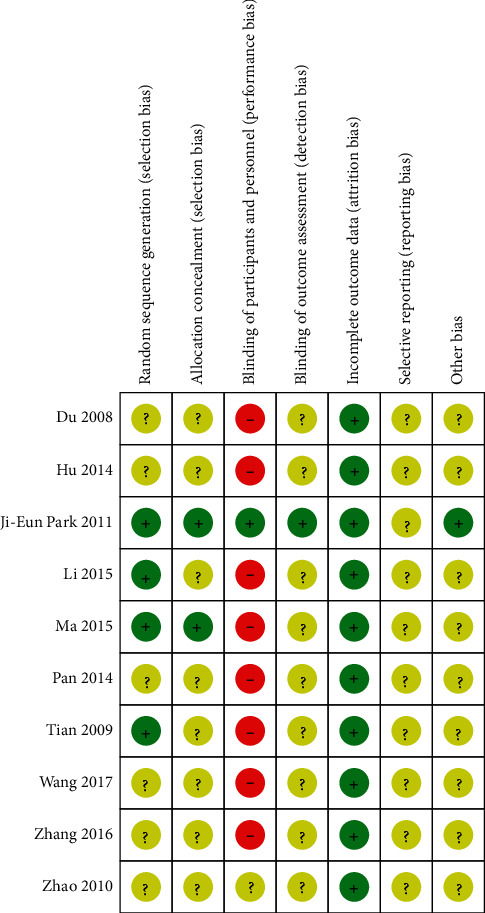
Risk of bias summary.

Table 2 and Figures 2 and 3 present the quality evaluations of the RCTs. Among the 10 articles, 4 provided a detailed description of the random grouping method, 6 only mentioned random grouping words but did not explain the random grouping method, and 2 performed concealment. Given that it was difficult to blind patients and implementers to moxibustion, only one study used blinding. One article earned a quality grade of A, and the nine other articles received a grade of B.

### 3.3. Outcome Analysis

#### 3.3.1. Clinical Effectiveness Rate

**Figure 4:**
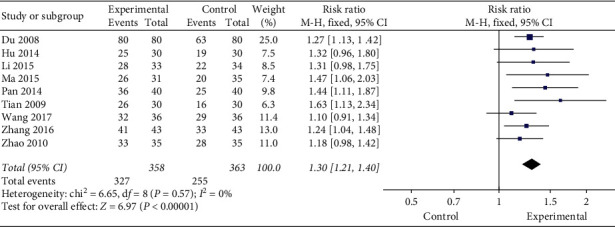
Meta-analysis of clinical effectiveness rate.

**Figure 5:**
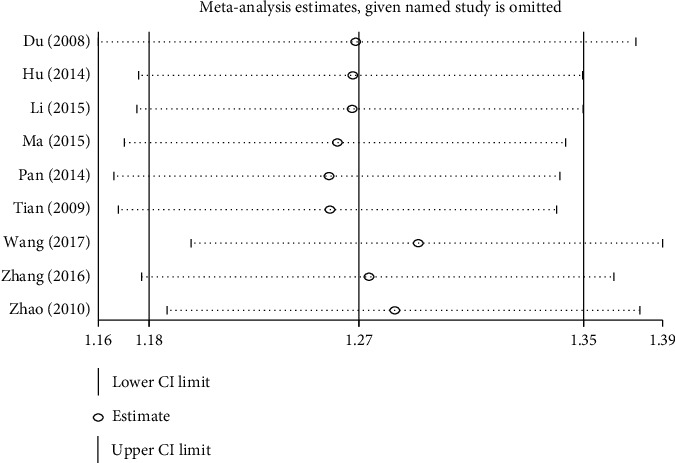
Sensitivity analysis of the effect of moxibustion on the clinical efficacy of constipation.

Of the 10 studies, nine determined the clinical effectiveness rate. Among them, 7 studies were evaluated using the Standards for Diagnosing Efficacy of TCM Syndrome, and 2 studies were conducted using the Guidelines for Clinical Research Guidance of New Drugs of TCM. A total of 721 patients were included. No statistical heterogeneity was found among the studies by using the *χ*^2^ test (*P*=0.57, *I*^*2*^ = 0%). Thus, the FEM was used. Results showed that compared with western medicine treatment or other Chinese medicine methods, moxibustion had evidently improved efficacy of constipation, and the difference was statistically significant (RR = 1.30, 95% CI [1.21, 1.40], *P* < 0.00001, Figure 4). Owing to the inconsistency of sample size and control interventions among studies, although there was no heterogeneity when the studies were combined, a sensitivity analysis was done in order to evaluate the stability of results. Research conclusions showed that sensitivity analysis did not appear to have a single study affecting the overall results, so the results were stable (Figure 5).

Due to the inconsistency of interventions in the combined articles, subgroup analyses of control group intervention, treatment course, and methods of moxibustion were conducted. No matter what kind of subgroup analysis, the conclusions were consistent with the above.

#### 3.3.2. Control Group Intervention

**Figure 6:**
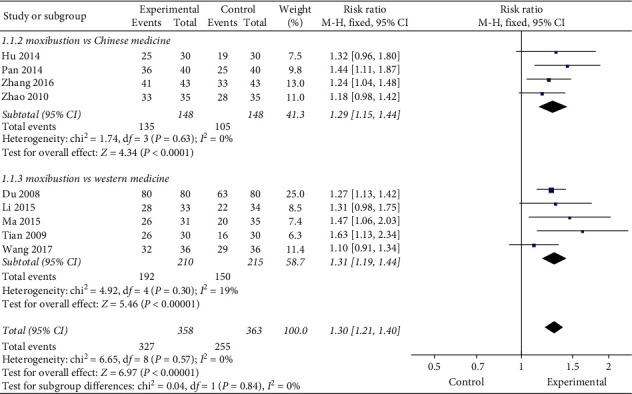
Subgroup analysis of different interventions.

According to the intervention measures of the control group, it could be divided into Chinese medicine treatment and western medicine treatment. The results showed that, compared with western medicine treatment or other traditional Chinese medicine methods, moxibustion was more clinically effective (RR = 1.31, 95% CI [1.19, 1.44], *P* < 0.00001; RR = 1.29, 95% CI [1.15, 1.44], *P* < 0.0001, Figure 6).

#### 3.3.3. Treatment Course

**Figure 7:**
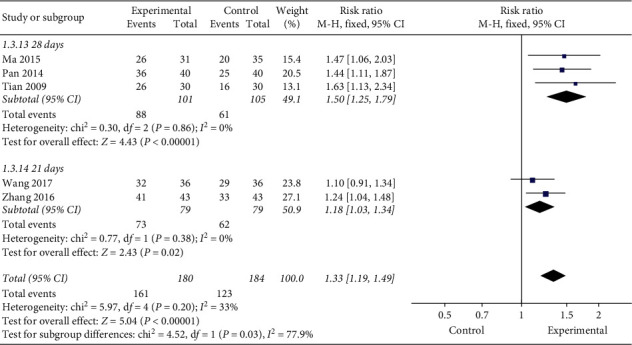
Subgroup analysis of different treatment courses.

In order to understand the effect of different treatment courses on clinical effectiveness rate, this study conducted a subgroup analysis of treatment course and found that the clinical effectiveness of moxibustion for 28 days (RR = 1.50, 95% CI [1.25, 1.79], *P* < 0.00001) was higher than that for 21 days (RR = 1.18, 95% CI [1.03, 1.34], *P*=0.02, Figure 7). However, when they were combined, there was some heterogeneity to a certain extent (*P*=0.2, *I*^2^ = 33%).

#### 3.3.4. Methods of Moxibustion

**Figure 8:**
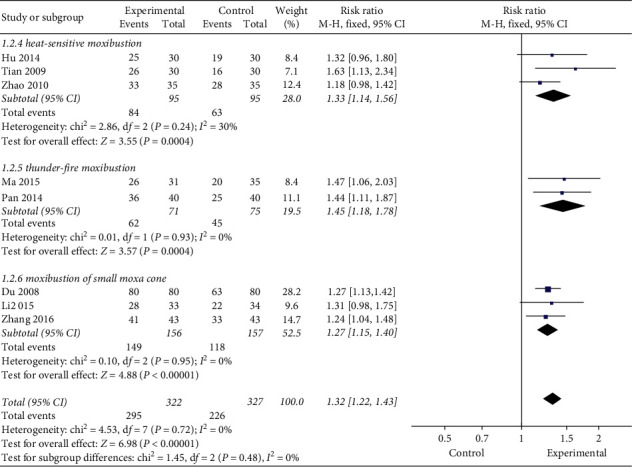
Subgroup analysis of different methods of moxibustion.

Similarly, we did a subgroup analysis of different moxibustion methods and found that, no matter what moxibustion method was used, the clinical effectiveness rate of moxibustion was higher than that of the control group (RR = 1.33, 95% CI [1.14, 1.56], *P*=0.0004; RR = 1.45, 95% CI [1.18, 1.78], *P*=0.0004; RR = 1.27, 95% CI [1.15, 1.40], *P* < 0.00001; Figure 8).

### 3.4. First Defecation Time

**Figure 9:**
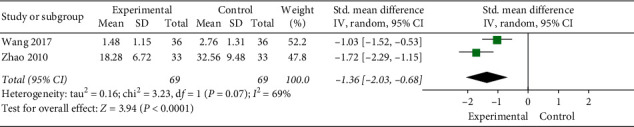
Meta-analysis of first defecation time.

Two studies compared the first defecation times of 138 patients. One study has evaluated the parameter in hours, and the other study evaluated the parameter in days. Thus, the standard mean difference was used. The *χ*^2^ test revealed statistical heterogeneity between the studies (*P*=0.07, *I*^*2*^ = 69%). Thus, the REM was used. Results showed that, compared with the control group, the moxibustion group had effectively shortened time of first defecation. The difference between the groups was statistically significant (SMD = −1.36, 95% CI [−2.03, −0.68], *P* < 0.0001, Figure 9).

### 3.5. Clinical Symptom Score

**Figure 10:**
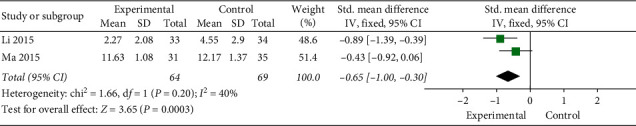
Meta-analysis of clinical symptom score.

Two studies compared the clinical symptom scores of 133 patients. The *χ*^2^ test showed no statistical heterogeneity among the studies (*P*=0.20, *I*^*2*^ = 40%). Thus, the FEM was used. Results showed that moxibustion can effectively improve the clinical symptoms of constipation (SMD = −0.65, 95% CI [−1.00, −0.30], *P*=0.0003, Figure 10).

### 3.6. BSS

**Figure 11:**
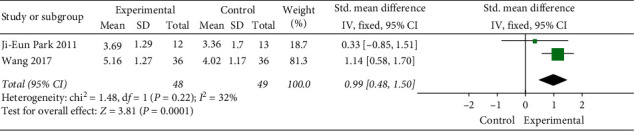
Meta-analysis of BSS.

Two studies used the Bristol score to assess the stool traits of 97 patients. The *χ*^2^ test revealed no statistical heterogeneity among the studies (*P*=0.22, *I*^*2*^ = 32%). Thus, FEM was used. Results showed that moxibustion can improve the patient's bowel traits (MD = 0.99, 95% CI [0.48, 1.50], *P*=0.0001, Figure 11).

### 3.7. Adverse Events

**Figure 12:**
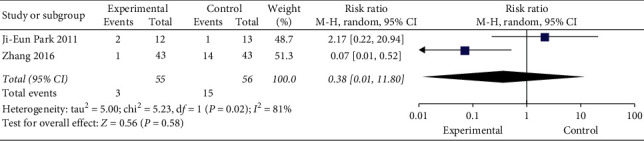
Meta-analysis of adverse event rate.

Of the 10 studies, four observed adverse effects after moxibustion, and one reported no evident adverse reaction. Furthermore, one study focused on adverse reactions but showed no statistical explanations and examples. Two studies reported the incidence of adverse reactions, as shown in Figure 12. The results showed that moxibustion therapy was as safe as other intervention methods (RR = 0.38, 95% CI [0.01, 11.80], *P*=0.58, Figure 12), so it was not yet possible to assess the safety level of moxibustion therapy.

### 3.8. Funnel Plot of Publication Bias

**Figure 13:**
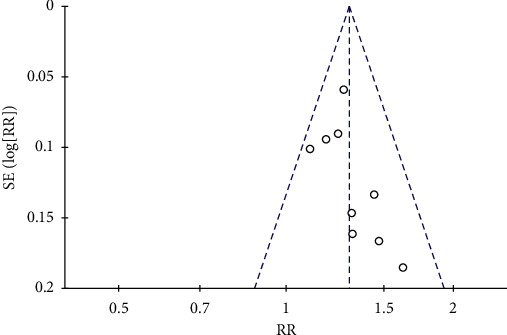
Funnel plot of publication bias.

As shown in Figure 13, a funnel chart analysis of clinical effectiveness rate was performed. Results showed that the clinical efficacy of constipation had a certain publication bias.

## 4. Discussion

### 4.1. Methodological Quality of Included Studies

Of the 10 included studies, 9 used clinical effectiveness rate, 2 reported first defecation time, 2 recorded clinical symptom scores, and 2 used BSS. This meta-analysis showed that moxibustion therapy was superior to other treatments in terms of clinical effectiveness, first defecation time, and improvement of symptoms.

Most studies use general, self-reported clinical efficacy as the main evaluation index and ignore observations of precise outcome indicators, such as colonic transmission tests and defecation frequency. Only one study reported defecation frequency. Results showed that moxibustion can increase defecation frequency. Thus, a combined analysis was not possible. In addition, the control group included in the study used different treatment methods to understand and evaluate the efficacy and safety of moxibustion for constipation in general.

### 4.2. Mechanism and Effect of Moxibustion for Constipation

Patients with constipation often combine multiple diseases, so their individual pathogenesis and etiology are still unclear. Pelvic floor muscle coordination disorders, enteric nervous system disease, unreasonable diet structure, lack of exercise, and depression may all cause constipation. The pathogenesis of constipation may be related to enteric nervous system disease, hormone neurotransmitters, interstitial cell distribution and function of Cajal, gastrointestinal motility, etc. At present, the mechanism of moxibustion for constipation has not been fully elucidated. The following are the three possible mechanisms. (1) Moxibustion can promote the acceleration of gastrointestinal motility by increasing the plasma content [33]. (2) Motilin (MTL) can promote gastrointestinal movement and improve gastrointestinal function. Moxibustion can significantly increase plasma MTL content after meals to promote gastrointestinal motility [34, 35]. (3) The mechanism by which moxibustion works may be related to its ability to improve the enteric nervous system and intestinal blood circulation [36].

The Chinese medicine believes that large intestinal conduction disorders lead to constipation, prolonged bowel cycle, and difficult drainage. Real heat; qi stagnation; and qi, yang, and blood deficiencies are the pathogenic mechanisms [37]. After the moxa stick is ignited, its medicinal properties and heat can enter the body through the acupoints on the body surface, which has the effects of warming and dispersing the cold, dredging the meridians, activating qi and blood, tonifying the yang, and preventing diseases [38]. Modern research [39] shows that the warm stimulation of moxibustion can promote gastrointestinal blood circulation and enhance gastrointestinal motility to promote defecation. Ten articles were included in this study. Although different moxibustion administration methods were conducted, the intervention time, course of treatment, and acupuncture points were used during the implementation. However, using moxibustion as a whole system will reveal that moxibustion therapy can significantly improve patients' constipation symptoms caused by multiple reasons. Two studies have also conducted a follow-up and found that moxibustion has significant long-term effects. However, a combined analysis was not conducted due to different follow-up times and observation indicators.

### 4.3. Different Conclusions of the Published Literature

Although studies have shown that moxibustion therapy has a good effect on constipation caused by various reasons [40–42], the conclusions are based on RCT trials of nonblind methods. However, a sample trial of sham moxibustion and related evidence show that moxibustion has no advantage in treating constipation [43]. Many explanations for the conclusion can be presented. The key factors may be that the type of syndrome in patients with constipation is not specific and the placebo of sham moxibustion and sample size are too small, which is also a possible direction for future studies. Some studies [44, 45] have pointed out that the choice of different acupoints and the compatibility of acupoints will influence the therapeutic effect; therefore, patients with different syndrome types and different basic diseases should choose different acupoints, and they cannot be generalized [46]. According to the meridian analysis of acupuncture points, this study shows that the frequency of application of the acupoints of stomach meridian of foot-yangming is the highest; correspondingly, Tianshu (ST25) and Zusanli (ST36) points are used frequently. Tianshu acupoints have the effect of regulating intestinal organs and regulating qi stagnation. Followed by “Sea of Yin Veins” Ren Mai, Guanyuan (RN4), Qihai (RN6), and Shenque (RN8) points are used more frequently. Acupoints of Ren Mai have the effect of warming the cold and the dispelling cold, nourishing yin and laxative. At present, studies have reviewed the treatment of constipation with acupuncture [47]. However, the efficacy and mechanism of moxibustion therapy alone have not been described. Therefore, evidence must be synthesized to evaluate the efficacy and safety of moxibustion therapy for constipation.

### 4.4. Limitations

The limitations of this evaluation system are as follows.

1. Given that moxibustion belongs to the category of traditional Chinese medicine, less foreign studies were found in this area. Only one English article was selected after screening. The methodological quality of the literature included in the comprehensive evaluation was not high. Except for one article having grade A, the quality of the studies was of grade B. These studies may cause bias, which may have affected the accuracy of the research conclusions to a certain extent. Furthermore, among four included detailed randomization methods, two achieved allocation concealment, and only one study blinded the patients and the assessors. Results showed a lack of scientific research methodology.
2. Some studies show a certain degree of heterogeneity when merged. The sources are caused by differences in acupuncture points, frequency, and course of treatment between the 10 included articles. The shortest course is 7 days, and the longest course is 56 days. The frequency is usually once a day but also includes every other day and 2-3 times a week. Intervention time also varied from 15 min to 60 min; these conditions may be related to the cause and duration of constipation.
3. Limited by the number and quality of included studies, this systematic review may have some bias. The small sample size and imperfect outcome indicators of most of the included studies may affect the argument intensity. For many indicators, such as “BSS” and “first defecation time,” only two studies can be analyzed, which may lead to insufficient extrapolation of results. Although most studies indicate that moxibustion is safe and has no side effects, this conclusion cannot be easily drawn from the results of this study. Thus, multicenter, large-sample, high-quality clinical RCTs should be carried out in the future.

## 5. Conclusions

This study shows that moxibustion has a certain effect on the management of constipation. However, it is not yet possible to assess the safety level of moxibustion therapy. In addition, the quality of the included literature is mostly low, and further rigorous studies are warranted.

## Figures and Tables

**Table 1:** Characteristics of the 10 trials identified in the literature search.

Included study	Syndrome pattern	Sample size		Intervention		Acupoints	Frequency	Treatment (days)	Outcome
		Experiment	Control	Experiment	Control				
Zhao et al. [23]	Yin deficiency and dryness	35	35	Moxibustion	Chinese medicine	BL25, BL32	30–60 min (qd)	7	1, 2, 6
Tian [24]	No description	30	30	Moxibustion	Mosabilli	BL23, BL25	40 min (qod)	28	1
Hu et al. [25]	No description	30	30	Moxibustion	Acupuncture	BL25, ST25	No (qd)	10	3
Yue and Li [26]	Qi deficiency	40	40	Moxibustion	Lactulose	RN6, RN4, RN8, ST25, ST36	20 min (qd)	28	1, 4
Pan et al. [27]	Yang deficiency	40	40	Moxibustion	Senna	ST25, RN8, RN6	20–30 min (qd)	28	1
Li et al. [28]	Deficiency of temper qi, deficiency of spleen and kidney yang	33	34	Moxibustion	Routine care	ST36, SP6	No (2–3 times/week)	56	3, 4
Wang [29]	No description	36	36	Moxibustion	Cisapilli	BL17, BL18, BL20、, BL21, BL22, BL23, BL25	60 min (qd)	21	1, 2, 5, 6
Du et al. [30]	No description	80	80	Moxibustion	Kaisell	ST30	No (qd)	3	1
Zhang [31]	No description	43	43	Moxibustion	Qi rong Runchang oral liquid	RN4	No (qd)	21	1, 6
Park et al. [32]	Qi deficiency or excess	12	13	Moxibustion	Sham moxibustion	ST23, ST27	No (3 times/week)	28	5, 6, 7, 8

1, clinical effectiveness rate: determined in accordance with the Chinese medicine industry standard “Diagnostic and Therapeutic Effect Standard of Chinese Medicine Disease Syndrome.” 2, first defecation time. 3, clinical effectiveness rate: formulated with reference to the Guiding Principles for Clinical Research of New Drugs in Traditional Chinese Medicine. 4, clinical symptom score. 5, Bristol stool form scale. 6, adverse reactions. 7, Constipation Assessment Scale (CAS). 8, defecation frequency.

**Table 2:** Risk of bias.

Included study	Random sequences	Allocation concealment	Blinding of subject and implementer	Blinding of outcome reporting	Complete data outcomes	Selective reporting of research results	Other sources of bias	Quality
Zhao et al. [23]	U	U	U	U	Y	U	U	B
Tian [24]	Y	U	N	U	Y	U	U	B
Hu et al. [25]	U	U	N	U	Y	U	U	B
Yue and Li [26]	Y	Y	N	U	Y	U	U	B
Pan et al. [27]	U	U	N	U	Y	U	U	B
Li et al. [28]	Y	U	N	U	Y	U	U	B
Wang [29]	U	U	N	U	Y	U	U	B
Du et al. [30]	U	U	N	U	Y	U	U	B
Zhang [31]	U	U	N	U	Y	U	U	B
Park et al. [32]	Y	Y	Y	Y	Y	U	Y	A

## Abbreviations

RCTs: Randomized controlled trials
TCM: Traditional Chinese medicine
BSS: Bristol stool form scale
FEM: Fixed effects model
REM: Random effects model
SMD: Standardized mean difference method
RR: Relative risk
CI: Confidence interval.
